# Patient Satisfaction with Pharmacist-Provided Health-Related Services in a Primary Care Clinic

**DOI:** 10.3390/pharmacy9040187

**Published:** 2021-11-21

**Authors:** Jacob N. Jordan, Thomas G. Wadsworth, Renee Robinson, Hayli Hruza, Amy Paul, Shanna K. O’Connor

**Affiliations:** 1 College of Health, University of Alaska Anchorage, Anchorage, AK 99508, USA; jakejordan@isu.edu; 2 College of Pharmacy, Idaho State University, Pocatello, ID 83209, USA; amy.paul@providence.org (A.P.); shannaoconnor@isu.edu (S.K.O.); 3 Whatcom County Health Department, Bellingham, WA 98225, USA; reneerobinson@isu.edu; 4 Providence Medical Group Primary Care Clinic, Anchorage, AK 99508, USA; hayli.hruza@gmail.com

**Keywords:** patient satisfaction, pharmacist, primary care, chronic disease, collaborations, medically underserved areas

## Abstract

(1) Background: Patient satisfaction plays an important role in the perceived value, sustained utilization, and coverage of healthcare services by payers and clinics. (2) Methods: A 33-question survey was designed to assess patient satisfaction and perceived value for healthcare services provided by a clinical pharmacist in a single primary care facility. It included general items from validated patient satisfaction surveys (i.e., PROMIS^®^, CAHPS) and pharmacist-specific items identified in selected literature. It was offered to all patients who were presenting for a new, unique visit with the clinical pharmacist at the medical clinic between May 2019 and April 2020. (3) Results: A total of 66 patients agreed to take the survey (RR = 100%), and the responses were overwhelmingly positive. However, men were more likely than women to report higher satisfaction (X2(1, n = 920) = 0.67, *p* = 0.027), and new patients reported higher satisfaction than existing patients (X2(1, n = 1211) = 1.698, *p* = 0.037). (4) Conclusions: The findings of this study indicate a high degree of patient satisfaction with pharmacist-provided healthcare services in the primary care setting.

## 1. Introduction

In the past 15 years, there has been an increased focus on the patient care experience and the involvement of patients in the evaluation and assessment of the healthcare provided through the use of patient-reported outcomes (PROs) and patient-reported outcome measures (PROMs) [1,2]. Patient satisfaction, a key global indicator of healthcare quality, plays an important role in measuring the value patients place on healthcare services, service utilization by patients, and coverage of services by payers [3]. Satisfied patients tend to continue using a valued service and better adhere to prescribed treatments, both of which ultimately lead to better health outcomes [4]. Other studies have explored the interaction between multiple dimensions of patient satisfaction on quality of healthcare services, including satisfaction with providers, interactions with clinicians, the medical facility, and leadership style [5,6,7].

Pharmacist-provided clinical health services address an often unmet primary care need, especially in rural and medically underserved communities [8,9,10,11]. Particularly in those settings, many pharmacists increasingly provide a variety of non-dispensing, clinical health services (e.g., medication management, chronic disease management, transitions of care, and preventative care services) improving healthcare access, service utilization, health outcomes, and quality of life [12,13]. Especially for rural patients, pharmacists are often the nearest and most accessible healthcare provider. However, the extent to which patients are satisfied with pharmacist-provided healthcare services in the primary care setting has been minimally studied for few conditions [14,15,16]. While the literature is robust supporting the strength of pharmacists’ contribution to the clinical care of patients, it is lacking when supporting the connection between patient satisfaction and pharmacist-provided healthcare services in the primary care setting, particularly in rural areas. It is therefore essential to critically evaluate this relationship, especially in light of shifts in healthcare reimbursement models towards quality. Consequently, the authors sought to contribute additional data related specifically to rural patient satisfaction in the primary care space. The objective of this study is to determine patient satisfaction with non-dispensing, general healthcare services rendered by a pharmacist in a primary care setting.

## 2. Materials and Methods

A survey was designed to assess patient satisfaction and perceived value for healthcare services provided by a clinical pharmacist embedded in a single primary care facility as a core healthcare provider between May 2019 and April 2020. The clinical pharmacist was established with the clinic three-years prior to the study period, has a number of trainings and certifications (post-graduate residency, Board Certified in Ambulatory Care Pharmacy (BCACP), Certified Diabetes Educator). Additionally, the pharmacist is enrolled and credentialed as a rendering provider with state and commercial insurance plans. The clinic practiced a Patient-Centered Medical Home model with care teams including physicians, nurse practitioners, physician assistants, pharmacists, nurses, and behavioral health professionals. The clinical pharmacist conducted scheduled visits with patients who were referred by other clinic providers for a specific purpose, including collaborative disease management of a variety of chronic conditions (i.e., diabetes, hyperlipidemia, anticoagulation, preventative health, osteoporosis); prescribing and adjusting medications; medication therapy management, administration, and medication counseling; and the administration of preventative healthcare services, such as smoking cessation and vaccinations. The survey was offered only to patients who were meeting with the pharmacist for the first time to establish care; this convenience-based sampling technique was determined to be appropriate given the resources available for the study and constraints specific to the practice setting. Patients were excluded from survey participation if they also had a same-day appointment with another clinic provider or were repeat visits with the same clinical pharmacist. Patients were also excluded if they were younger than 18 years of age or non-English speaking. All visits were documented, coded, and billed in the clinic’s electronic health record in the same fashion as other core healthcare providers.

Investigators reviewed literature and institution-specific documents for existing patient satisfaction or perception surveys to inform survey design. The final survey design included general items from validated patient satisfaction surveys (i.e., PROMIS^®^, CAHPS) and pharmacist-specific items identified in selected literature [2,17]. The result was a 33-item survey consisting of 15 questions related to demographics and service utilization, and 18 questions focused on patient satisfaction. The patient satisfaction questions were divided into four key domains: (1) Patient Experience, (2) Self-Efficacy for Managing Medications and Treatment, (3) Perceived Value of Pharmacist-Provided Health Services, and (4) Willingness to Pay for those services.

The survey items selected to capture Self-Efficacy for Managing Medications were specifically adapted from PROMIS Self-Efficacy for Managing Medications and Treatment [2,17]. All other survey components were selected and revised based on relevance to pharmacist healthcare services, suggestions received from the clinic pharmacist, and face validity from patients and colleagues. A five-point Likert scale was used to assess respondent perceptions, ranging from the negative “Strongly disagree” to the positive “Strongly agree”.

The researchers selected the following 18-survey items to express the four domains of patient satisfaction for analysis (see Table 1):

Recruitment for survey participation occurred before or after a patient’s scheduled appointment with the clinical pharmacist at the clinic. At the completion of the visit, the pharmacist recorded key metrics, including visit type, CPT codes, length of visit, and the specific services provided. The patient survey was then administered via electronic tablet or paper copy to consenting patients. Investigators used a script to recruit eligible patients, and a consent form was provided with the questionnaire. Patients were informed that the purpose of the survey was to assess patient satisfaction, participation was voluntary, and survey responses would be anonymous. The study was reviewed and approved by the university and medical center institutional review boards.

Likert responses were tabulated according to the four survey domains. Results were analyzed using descriptive statistics and evaluated for each domain and overall patient satisfaction. A chi-square test of independence was performed to determine if there was an association between select patient factors [r1] (i.e., gender, patient status, age, and payor type) and participant patient satisfaction. For the chi-square analysis, the Likert responses of ‘1’ and ‘2’ were combined to represent ‘unsatisfied’; responses ‘4’ and ‘5’ were aggregated to convey ‘satisfied’; and neutral responses were excluded.

## 3. Results

Sixty-six participants were asked to complete the survey, and all agreed to participate (100% participation). A total of 59 participants submitted a complete post-visit survey (89.4% response rate for complete surveys). Seven participants did not fully complete the survey; however, all responses are considered in the analyzed data and contribute to the calculated percentages. Thirty-six participants identified as female (35.4%), 23 as male (35.4%), and six as other (9.2%). Nineteen patients were new to the clinic (29.2%), while 46 patients identified as ‘existing’ patients who were established at the clinic (70.8%). Most participants were 65 years or older (72.6%, n = 45), followed by those 45 to 64 years (14.5%, n = 9), 30 to 44 years (8.1%, n = 5), and those 18 to 29 years of age comprised the smallest age group (4.8%, n = 3).

The average length of the surveyed visits was 38 min (sd = 19.15 min). Visit activities included medication education (94%), disease education (62%), comprehensive medication management (55%), care coordination (42%), medication initiation (37%), and medication administration (34%). The most common purpose for the visit was diabetes management, followed by lipid management, hypertension management, anticoagulation, osteoporosis management, and other preventive health services.

**Figure 1:**
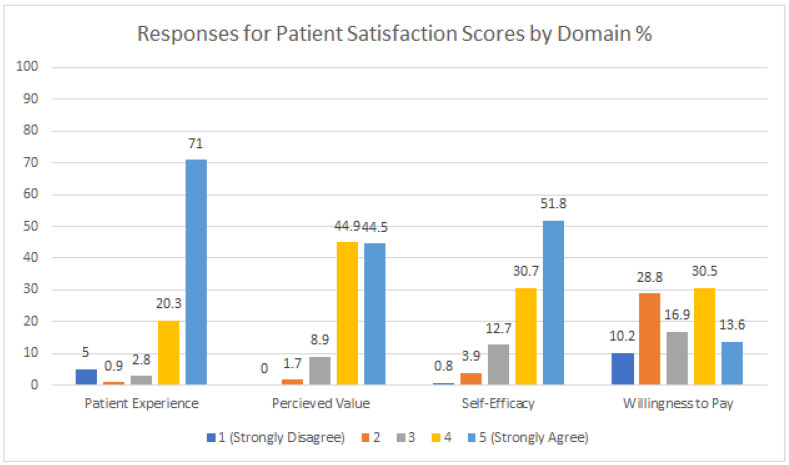
Aggregate patient satisfaction scores by domain.

The aggregate of patient satisfaction scores was overwhelmingly positive, with the majority of responses marked as ‘strongly agree’ or ‘agree’ (85.6%), and a minority of responses marked a two for ‘disagree’ or a one for ‘strongly disagree’ (6.4%, n = 71). Patient satisfaction by the survey domains indicated participants selected ‘strongly agree’ most often for the ‘Patient Experience’ domain (71%) and ‘Self-Efficacy’ domain (51.8%). The ‘Perceived Value’ domain was also positive. The ‘Willingness to Pay’ domain displayed a more regular distribution across the Likert categories and contained the largest percentage of ‘strongly disagree’ (10.2%) and ‘disagree’ (28.8%) responses than any of the other survey domains (Figure 1).

**Figure 2:**
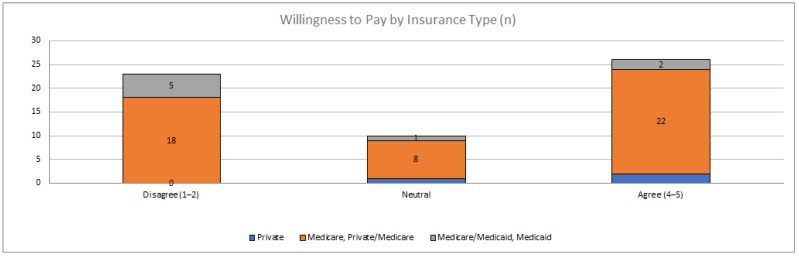
Aggregate Likert responses for ‘Willingness to Pay’ by insurance type.

While there was no statistically significant relationship between overall patient satisfaction with payor type, it is notable that of all respondents marking ‘strongly disagree’ or ‘disagree’ in the ‘Willingness to Pay’ domain (n = 22) had Medicare or Medicaid (38%). The responses to the ‘Willingness to Pay’ domain broken down by payer type are presented in Figure 2.

There was no statistically significant relationship between survey responses and age or payor type (see Table 2).

However, men were more likely than women to report higher satisfaction (X2(1,N = 920) = 0.67, *p* = 0.027), and new patients reported higher satisfaction than existing patients (X2(1,N = 1211) = 1.698, *p* = 0.037).

## 4. Discussion

Patient satisfaction is an important and commonly used indicator of healthcare quality and, in part, establishes the value of those services [2,18,19,20,21]. This study utilized validated patient satisfaction survey instruments (i.e., PROMIS^®^) augmented by pharmacist-specific items identified in selected literature and demonstrated a high degree of overall patient satisfaction with general chronic disease and medication management services received from a primary care pharmacist. Specifically, survey participants were highly satisfied with the experience and expressed greater confidence and self-efficacy in managing medications and/or treatments. These results are consistent with other reported satisfaction metrics (timeliness and efficiency) regarding specialty care services delivered by pharmacists and other healthcare providers in a variety of clinical settings [21].

Patients in our study also expressed a high degree of value and willingness to pay for the services provided by the clinical pharmacist, which are perhaps the more compelling indicators of their satisfaction [22].

The survey has several limitations. First, the source population may not be representative of the target population (i.e., primary care patients) and may only reflect the experiences in a single health system and single clinical pharmacist. Further investigation is required to assess the generalizability of our findings and to make additional connections with the theory behind patient satisfaction relationship to healthcare quality and outcomes. Next, we did not directly compare patient satisfaction with other provider types, clinics, or health outcomes including quality. Regardless, this does not diminish the overwhelmingly positive satisfaction of the patients receiving healthcare services from a primary care pharmacist—this data may be useful for other rural clinics interested in incorporating pharmacist-provided healthcare services or for researchers interested in exploring the impact of pharmacists on patient satisfaction. Finally, we did not analyze the effect the type of service provided may have had on patient satisfaction. This information would be helpful to further differentiate and understand the value of particular healthcare services provided by clinical pharmacists.

## 5. Conclusions

This study demonstrated a high degree of patient satisfaction with general chronic disease and medication management services received from a primary care pharmacist. The comparative interaction of patient satisfaction with other healthcare disciplines, care teams, or health outcomes requires further investigation. The results of this study add to the growing body of knowledge about the contribution of clinical pharmacist services to patient-centered care.

## Figures and Tables

**Table 1:** Domains and factors of patient satisfaction.

Domain	Factors *
Patient Experience	(a) ease of making an appointment with pharmacist compared to other healthcare providers (b) time allotted to ask pharmacist questions (c) pharmacist was approachable (d) pharmacist addressed my concerns (e) provider knew important information about medical and treatment history (f) pharmacist used words I could understand (g) pharmacist provided instructions on how to take medications (h) pharmacist provided information on side effects of my medications
Self-Efficacy for Managing Medications and Treatment [1]	(a) when and how to take medication (b) managing medications without help (c) remembering to take medications (d) participation in medication decisions (e) confidence in ability to manage health condition (f) ability to follow a medication treatment plan
Perceived Value	(a) The pharmacist is an essential and effective part of my healthcare team. (b) The pharmacist services I received today are a valuable part of my healthcare. (c) My healthcare would be diminished (reduced) if I did not receive these services today. (d) I would have trouble taking care of my condition without the services I received today.
Willingness to Pay	(a) I would be willing to pay (out-of-pocket) for the services I received today if they were not covered by my health insurance plan.

* (Rated on 5-pt Likert scale (1 = Strongly disagree, 5 = Strongly agree).

**Table 2:** Overall patient satisfaction score by demographic feature.

Demographics.	Strongly Disagree/Disagree	Strongly Agree/Agree
**Gender**		
Male	3.0 (13)	97.0 (421)
Female	8.9 (65)	91.1 (669)
Other	0 (0)	100 (43)
**Patient Status**		
New	4.9 (16)	95.1 (309)
Existing	7.0 (62)	93.0 (824)
**Age**		
18–29	0.0 (0)	100 (45)
30–44	1.3 (1)	98.7 (77)
45–64	4.6 (7)	96.4 (145)
65 and Older	7.4 (19)	92.6 (874)
**Payor**		
Private Only	0.0 (0)	100 (2)
Medicare, Private/Medicare	45 (18)	55 (22)
Medicare/Medicaid, Medicaid	71.4 (5)	28.6 (2)

Proportion of Likert response within select demographic feature. Results reported as percentage of Likert response ‘Strongly disagree’ and ’Disagree’ or ’Agree’ and ’Strongly disagree’ (n).

## Data Availability

This does not apply.

## References

[1] Weldring T., Smith S.M.S. (2003). Patient-Reported Outcomes (PROs) and Patient-Reported Outcome Measures (PROMs). Health Serv. Insights.

[2] Cella D., Riley W., Stone A., Rothrock N., Reeve B., Yount S., Amtmann D., Bode R., Buysse D., Choi S. (2010). The Patient-Reported Outcomes Measurement Information System (PROMIS) developed and tested its first wave of adult self-reported health outcome item banks: 2005–2008. J. Clin. Epidemiol..

[3] Garman A.N., Garcia J., Hargreaves M. (2004). Patient satisfaction as a predictor of return-to-provider behavior: Analysis and assessment of financial implications. Qual. Manag. Healthc..

[4] Oluwole E., Osibogun O., Adegoke O., Adejimi A., Adewole A., Osibogun A. (2019). Medication adherence and patient satisfaction among hypertensive patients attending outpatient clinic in Lagos University Teaching Hospital, Nigeria. Niger. Postgrad. Med. J..

[5] Jameel A., Asif M., Hussain A., Hwang J., Bukhari M.H., Mubeen S., Kim I. (2019). Improving patient behavioral consent through different service quality dimensions: Assessing the mediating role of patient satisfaction. Int. J. Environ. Res. Public Health.

[6] Asif M., Jameel A., Sahito N., Hwang J., Hussain A., Manzoor F. (2019). Can leadership enhance patient satisfaction? Assessing the role of administrative and medical quality. Int. J. Environ. Res. Public Health.

[7] Hussain A., Asif M., Jameel A., Hwang J. (2019). Measuring OPD patient satisfaction with different service delivery aspects at public hospitals in Pakistan. Int. J. Environ. Res. Public Health.

[8] Murphy P.A., Frazee S.G., Cantlin J.P., Cohen E., Rosan J.R., Harshburger D.E. (2012). Pharmacy provision of influenza vaccinations in medically underserved communities. J. Am. Pharm. Assoc..

[9] Como M., Carter C.W., Larose-Pierre M., O’Dare K., Hall C.R., Mobley J., Robertson G., Leonard J., Tew L. (2020). Pharmacist-led chronic care management for medically underserved rural populations in Florida during the COVID-19 pandemic. Prev. Chronic Dis..

[10] Le L.D., Paulk I.R., Axon D.R., Bingham J.M. (2021). Comprehensive medication review completion in medically underserved areas and populations. J. Health Care Poor Underserved.

[11] Blazejewski L., Vaidya V., Pinto S., Gaither C. (2013). Pharmacists’ perceived barriers providing non-dispensing services to underserved populations. J. Community Health.

[12] Pande S., Hiller J.E., Nkansah N., Bero L. (2013). The effect of pharmacist-provided non-dispensing services on patient outcomes, health service utilisation and costs in low- and middle-income countries. Cochrane Database Syst. Rev..

[13] Nkansah N., Mostovetsky O., Yu C., Chheng T., Beney J., Bond C.M., Bero L. (2010). Effect of outpatient pharmacists’ non-dispensing roles on patient outcomes and prescribing patterns. Cochrane Database Syst. Rev..

[14] Sherrill C.H., Cavanaugh J., Shilliday B.B. (2017). Patient satisfaction with Medicare annual wellness visits administered by a clinical pharmacist practitioner. J. Manag. Care Spec. Pharm..

[15] Martin M.T., Faber D.M. (2016). Patient satisfaction with the clinical pharmacist and prescribers during hepatitis C virus management. J. Clin. Pharm. Ther..

[16] Hatton J., Chandra R., Lucius D., Ciuchta E. (2018). Patient satisfaction of pharmacist-provided care via clinical video teleconferencing. J. Pharm. Pract..

[17] Consumer Assessment of Healthcare Providers and Systems (CAHPS) Home Page. https://www.ahrq.gov/cahps/index.html.

[18] Chen Q., Beal E.W., Okunrintemi V., Cerier E., Paredes A., Sun S., Olsen G., Pawlik T.M. (2019). The association between patient satisfaction and Patient-Reported Health Outcomes. J. Patient Exp..

[19] Agosta L.J. (2009). Patient satisfaction with nurse practitioner-delivered primary healthcare services. JAANP.

[20] Hooker R.S., Moloney-Johns A.J., McFarland M.M. (2019). Patient satisfaction with physician assistant/associate care: An international scoping review. Hum. Resour. Health.

[21] Ismail A., Gan Y.N., Ahmad N. (2020). Factors associated with patient satisfaction towards pharmacy services among out-patients attending public health clinics: Questionnaire development and its application. PLoS ONE.

[22] Nguyen E., Walker K., Adams J.L., Wadsworth T., Robinson R. (2021). Reimbursement for pharmacist-provided health care services: A multistate review. J. Am. Pharm. Assoc..

